# Extensive penile glans and corpus spongiosum necrosis due to catheter traction following laparoscopic radical cystoprostatectomy: a rare case report and literature review

**DOI:** 10.1186/s12894-023-01289-4

**Published:** 2023-07-10

**Authors:** Mehmet Akyuz, Ridvan Kayar, Abdikarim Hussein Mohamed

**Affiliations:** Department of Urology, Health Sciences University HaydarpasaNumune Training and Research Hospital, İstanbul, Turkey

**Keywords:** Penile glans, corpus spongiosum necrosis, Penile preservation

## Abstract

**Background:**

Penile glans and corpus spongiosum necrosis is an extremely rare urologic condition associated with substantial morbidity.

**Case presentation:**

We report a rare case presenting extensive penile glans and corpus spongiosum necrosis following catheter traction in a 71-year-old male patient who had a laparoscopic radical cystoprostatectomy for muscle-invasive bladder cancer. The patient has no preexisting diabetes mellitus or chronic renal failure. The case was successfully managed with penile preservation. During the procedure, it was observed that the necrosis was not limited to the glans. The necrosis had spread to the entire penile urethra and corpus spongiosum, and an excision of approximately 14 cm of corpus spongiosum was performed.

**Conclusion:**

This is the first case presenting extensive length of penile glans and corpus spongiosum necrosis managed successfully with penile preservation, enabling reaching the best functional and esthetic results reported in the literature. Early detection and urgent imaging with a high index of suspicion ensure a favorable outcome. The main treatment steps are careful evaluation, appropriate therapy, and prompt intervention depending on the severity.

## Introduction

Penile glans and corpus spongiosum necrosis is an extremely rare and underreported urologic condition associated with substantial morbidity and mortality [1]. It is mostly associated with poorly controlled diabetes mellitus, end-stage renal disease due to calciphylaxis, obesity, priapism, venous outlet obstruction, systemic vasculitis, and IV drug use [2].

Ischemia of the penis is an uncommon condition due to the superior collateral arteries despite the paired internal pudendal arteries, which are branches of the internal iliac arteries [3].

Its rarity, combined with likely understudying, optimal treatment has not yet been established. However, several reports utilized various treatment options of non-guideline-based management, with most of these cases managed with partial or total penectomy [4]. We report a rare case presenting extensive penile glans and corpus spongiosum necrosis following catheter traction in a 71-year-old male patient who had a laparoscopic radical cystoprostatectomy for muscle-invasive bladder cancer. The case was successfully managed with penile preservation, excision of the necrotic glans and corpus spongiosum, and appropriate antimicrobial therapy.

## Case report

**Fig. 1:**
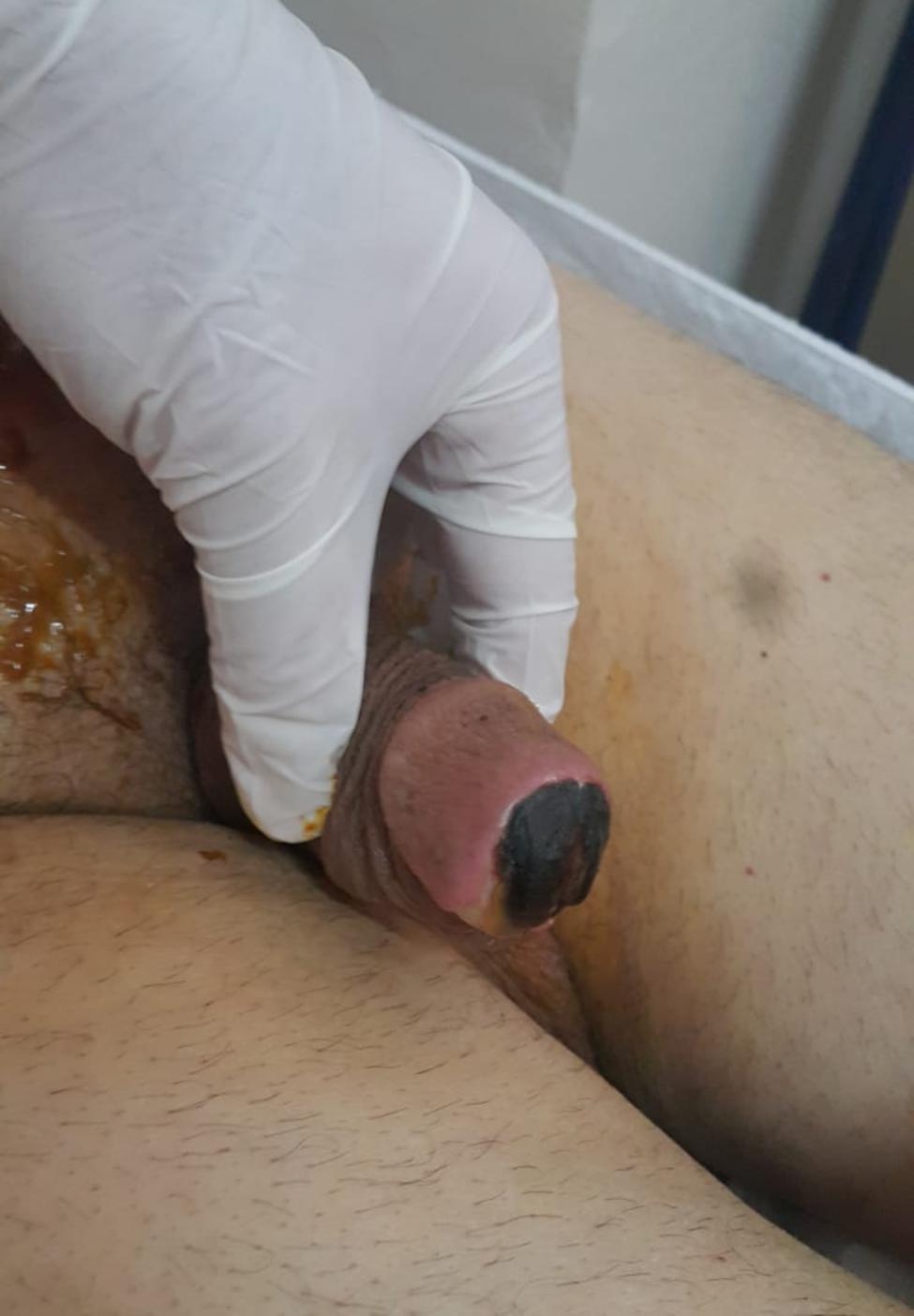
Penile glans necrosis

The present case is a 71-year-old male patient who had a laparoscopic radical cystoprostatectomy and ileal conduit for muscle-invasive bladder cancer (pathological stage T4aN2Mx). During the perioperative period, a foley catheter was used as traction and as a drain through the urethral route, and the catheter was removed on the 4th postoperative day. The patient’s past medical history does not reveal chronic disease, including diabetes. Also, his renal function test and other laboratory parameters were within normal limits. The preoperative clinical lymph node status was N0. The intraoperative blood loss was minimal, and no blood transfusion was needed. Enoxapirin was used for one month postoperatively. Two weeks postoperatively, the patient presented with yellow-white discharge from the meatus and bruising in the glans around the meatus (Fig. 1). Physical examination revealed glandular necrosis. Appropriate antimicrobial therapy (Meropenem + Teicoplanin + clindamycin ) was initiated after the patient’s admission. Initial laboratory investigation revealed an elevated white blood cell count (12,420ng/dl), and CRP (19.9), while other parameters were within normal limits.

The urethral discharge culture showed an E.coli, Klebsiella pneumonia, and Enterococcus faecium. Colistin and Tigecycline antimicrobial therapy were started after infectious disease consultation.

**Fig. 2:**
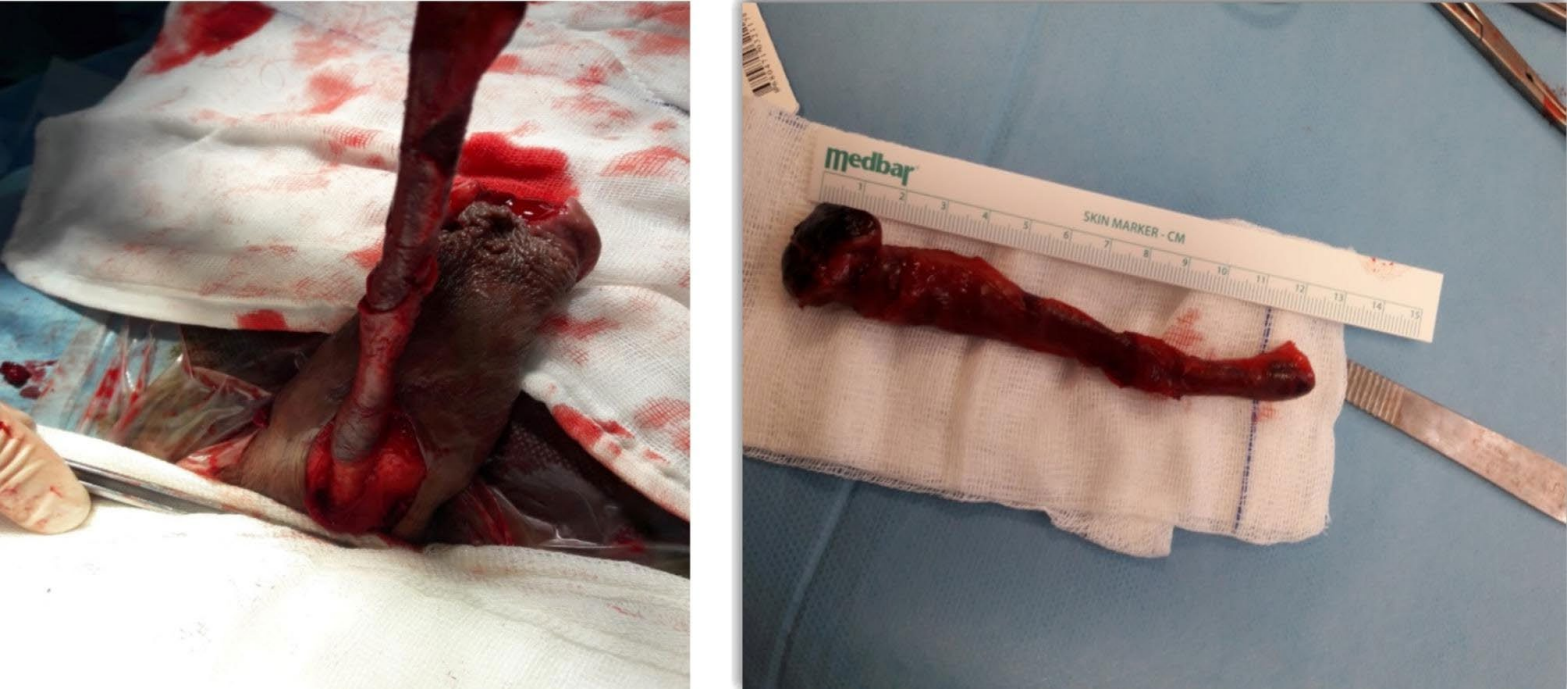
Intraoperative findings revealed an extensive Corpus spongiosum necrosis and excision of approximately 14 cm up to healthy tissue

Surgical exploration was planned for the patient, as there was no improvement on the 9th day of the antibiotic therapy. During the procedure, it was observed that the necrosis was not limited to the glans. The necrosis had spread to the entire penile urethra and corpus spongiosum, and an excision of approximately 14 cm of corpus spongiosum was performed (Fig. 2).

**Fig. 3:**
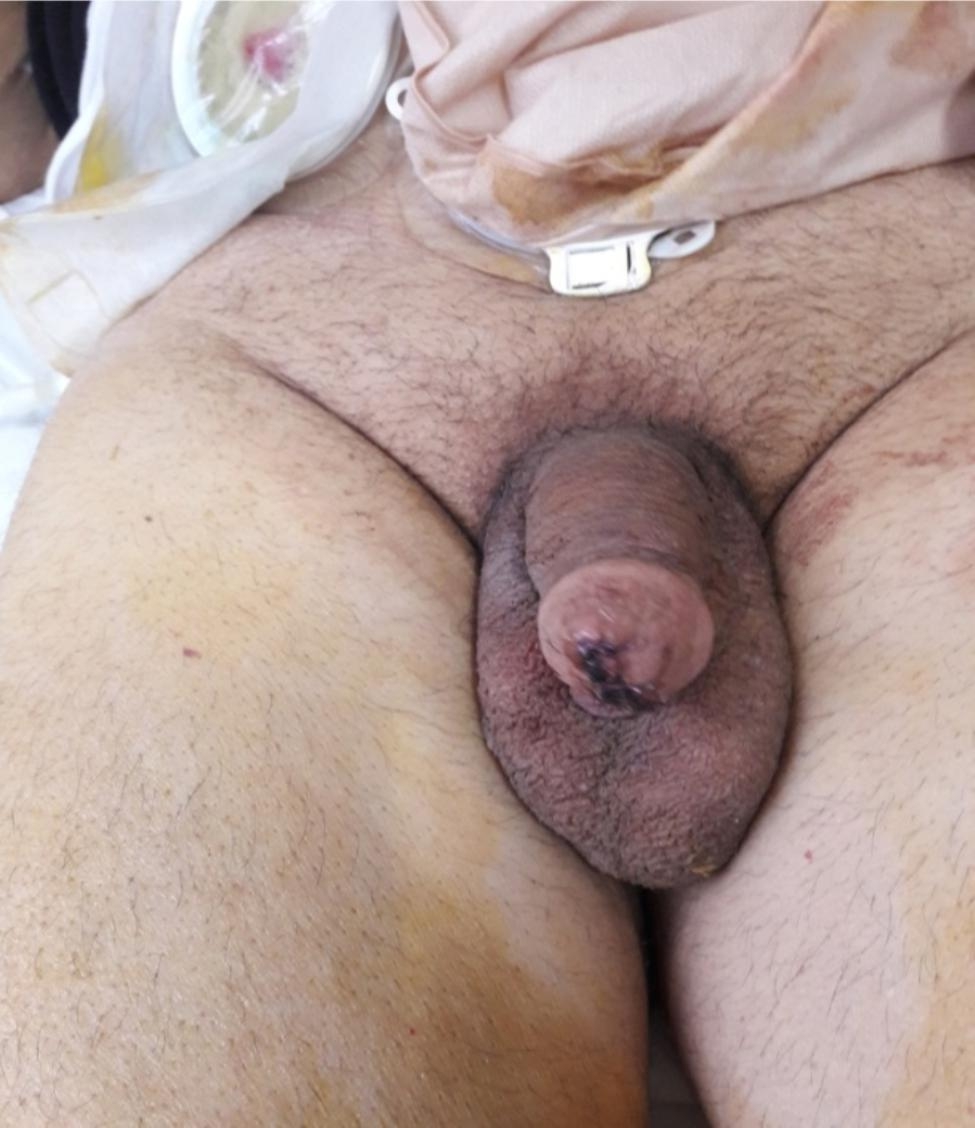
Postoperative view of the preserved penis

The postoperative period was uneventful, and the patient was discharged on the 6th postoperative day (Fig. 3). The pathology findings reported no malignancy and showed dıffuse hemorrhage and necrosıs of the urethra and extendıng to the surrounding tıssues. Glans necrosıs, ulceratıon, hemorrhage and chronıc ınflammatıon were also reported.

## Discussion

Penile necrosis is a very rare and devastating pathology that can result in partial or total penectomy in most cases. A deep search in the literature identified one case report of glans necrosis following traction of the urethral catheter in a 69-year-old man who had undergone transurethral resection of the prostate for benign prostatic hyperplasia. It was confined to the glans and was managed conservatively on antimicrobial therapy and watchful waiting until the gangrenous part sloughed off, leaving a defect on the glans penis [5]. To our knowledge, our case is the second case of penile glans necrosis following catheter traction reported in the literature. Our case underwent a major operation which predisposes diminished blood supply, and compression of the dorsal vein complex from the traction of the catheter may block the retrograde flow of the blood, not only in the glans; we also think that it causes necrosis in the entire penile urethra. Necrosis spreads rapidly towards the bulbar urethra due to ischemia and weakening of the local defense following the deterioration of vascularization and bacterial colonization from the catheterization. Early detection and urgent imaging with a high index of suspicion ensure a favorable outcome. Pelvic MRI or CT to exclude abscess or compression form haematoma, penile MRI and even angiography could be useful for the differential.

The laparoscopic approach before open surgery could be performed safely for bladder cancer as well as many other surgical diseases. Laparoscopic minimally invasive surgery should be preferred to open surgery due to its advantages. However, both open surgery and laparoscopic surgery have their set of pros and cons [6].

Akagiet al. reported a case of complete necrosis of the corpus spongiosum and corpora cavernosa after repeated transurethral surgical procedures. They managed with total penectomy and perineal urethrostomy [7]. Compared to our case, our patient had an intact corpora cavernosa despite presenting an extensive length of penile glans and corpus spongiosum necrosis. It was managed successfully with penile preservation, enabling the best functional and esthetic results.

In the few cases reported in the literature, there are different causes related to glans or penile necrosis and a wide diversity of treatment approaches.

Vascular occlusive pathologies are the leading cause of glans and penile necrosis. It is mainly associated with renal failure, diabetes, and venous thromboembolic diseases [8]. In end-stage renal failure, as a result of the occlusion of increased calcium and phosphate products, especially in the intima of small and medium-sized vessels, associated with secondary hyperparathyroidism, it causes well-circumscribed plaque, necrosis, and ulceration in the glans due to insufficient vascular supply in the tissue. In a review of 15 Japanese patients with calciphylaxis-related necrosis of the penis, the mainstay management was partial penectomy for 11 patients and total penectomy in the remaining four cases [8]. The authors reported a poor prognosis with calciphylaxis-related necrosis. Kharbach and colleagues reported a 54-year-old patient with idiopathic necrosis of the glans penis managed with partial penectomy [9]. Agustino and Irdam reported a 30-year-old male with ischemic related to aortic dissection who presented with acute limb ischemia, necrosis of the penis, and scrotum gangrene managed with debridement of the penis and scrotum and urinary diversion with percutaneous cystostomy [3].

Very few reports mention the use of angiography for the diagnosis of penile necrosis. Shah and colleagues reported a known case of hypertension, diabetes, and chronic renal failure who underwent circumcision for phimosis and developed gangrene of the glans penis two weeks later. The patient underwent internal pudendal artery angioplasty and a partial penectomy [10]. Kim et al. reported unexpected penile glans ischemic necrosis after internal pudendal arterial embolization in a patient with a post-traumatic internal pudendal artery-urethral fistula [11]. The authors reported successful treatment of the patient with intravenous infusion of alprostadil, oral pentoxifylline, and tadalafil. In a case series of six patients with penile glans necrosis (PGN) following prostatic artery embolization (PAE) due to failed medical benign prostate hyperplasia (BPH) therapy [12]. The authors proposed the role of hyperbaric oxygen therapy (HBOT) for PGN. HBOT appears to accelerate recovery with better erection scores than conservative methods.

Penile strangulation and gangrene may follow a condom catheter frequently used to manage male urinary incontinence, as reported by Zaghbib and associates [13]. Even if it’s rare, it has devastating complications, so care should be given to debilitated and psychiatric populations.

## Conclusion

This is the first case presenting extensive length of penile glans and corpus spongiosum necrosis managed successfully with penile preservation, enabling reaching the best functional and esthetic results reported in the literature. Early detection and urgent imaging with a high index of suspicion ensure a favorable outcome. The main treatment steps are careful evaluation, appropriate therapy, and prompt intervention depending on the severity.

## Data Availability

Data included in the manuscript.

## Abbreviations

BPH: benign prostate hyperplasia
HBOT: hyperbaric oxygen therapy
PGN: penile glans necrosis
PAE: prostatic artery embolization
